# Approaches to Daily Monitoring of the SARS-CoV-2 Outbreak in Northern Italy

**DOI:** 10.3389/fpubh.2020.00222

**Published:** 2020-05-22

**Authors:** Giovenale Moirano, Lorenzo Richiardi, Carlo Novara, Milena Maule

**Affiliations:** ^1^Department of Medical Sciences, University of Turin, and CPO Piemonte, AOU Città della Salute e della Scienza, Turin, Italy; ^2^Department of Electronics and Telecommunications, Politecnico di Torino, Turin, Italy

**Keywords:** epidemiology, COVID-19, public health, infectious disease, outbreak analyses

## Abstract

Italy was the first European country affected by the Sars-Cov-2 pandemic, with the first autochthonous case identified on Feb 21st. Specific control measures restricting social contacts were introduced by the Italian government starting from the beginning of March. In the current study we analyzed public data from the four most affected Italian regions. We (i) estimated the time-varying reproduction number (*R*_*t*_), the average number of secondary cases that each infected individual would infect at time *t*, to monitor the positive impact of restriction measures; (ii) applied the generalized logistic and the modified Richards models to describe the epidemic pattern and obtain short-term forecasts. We observed a monotonic decrease of *R*_*t*_ over time in all regions, and the peak of incident cases ~2 weeks after the implementation of the first strict containment measures. Our results show that phenomenological approaches may be useful to monitor the epidemic growth in its initial phases and suggest that costly and disruptive public health controls might have had a positive impact in limiting the Sars-Cov-2 spread in Northern Italy.

## Introduction

With an increasing number of cases throughout the world, on the 11th of March WHO declared COVID-19 a pandemic and called for governments to take urgent and aggressive actions (1). Italy was the first European country affected by local transmission of Sars-Cov-2. The first confirmed autochthonous COVID-19 case in Italy was identified on Feb. 21st (2), followed by the detection of clusters of cases in 11 relatively small municipalities (10 in Lombardy and 1 in Veneto). On February 22nd, the Italian government introduced quarantine on more than 50,000 people from the 11 municipalities. Despite this prompt reaction, 1 week later, the number of cases had reached 650 (3). On March 8th, Italy became the second most affected country in the world, after China (4). In order to contain the SARS-CoV-2 burden on the national health system, specific measures restricting social contact were first introduced in the northern regions, where most cases had occurred, then extended to the whole country on March 9th. These measures were further tightened on March 21st: all Italian businesses were closed, with the exception of those essential to the country's supply chains.

In the early phases of an outbreak, epidemiological data is limited and the parameters necessary to inform and calibrate mechanistic transmission models may be difficult to estimate. It is, however, crucial to monitor the pattern of epidemic growth, whilst incorporating uncertainty, in order to understand the current evolution of the outbreak and provide an early assessment of the potential impact restrictive measures.

With the current study, we have analyzed public data from the four most affected Italian regions (Lombardy, Veneto, Emilia Romagna, Piedmont) using approaches suitable to the initial phases of an epidemic, which could help the day-by-day monitoring and the decision-making process.

We estimated the time-varying reproduction number and used the generalized logistic growth model and the generalized modified Richards model to characterize the early behavior of the epidemic. These approaches have been used and validated in previous epidemics and applied to the recent SARS-CoV-2 epidemic in China and national data from other countries (5–7, 18).

## Methods

Daily counts of new infections and deaths, to April 30th, were computed from data available from the website of the Italian Ministry of Health/Civil Protection (3).

### Monitoring of Time-Varying Reproductive Number

The time-varying reproductive number, *R*_*t*_, is the average number of secondary cases that each infected individual would infect if the conditions remained as they were at time t (8). Typically, *R*_*t*_ decreases over time starting from *R*_0_, the basic reproductive number, as a consequence of both the depletion of susceptible individuals and effective control efforts (9). A monotonic decrease of *R*_*t*_ over time may indicate the positive impact of measures introduced to control the epidemic; whereas an unstable behavior or a sudden growth of *R*_*t*_ may suggest that corrective or additional measures are necessary. We estimated *R*_*t*_ using the Epi-Estim package in the R software environment (10), according to the following equation:

$$\begin{array}{l} I_{t}=R_{t\;}\overset{t}{\underset{s=1}{\sum }}{I_{t-s}w}_{s}, \end{array}$$

where *I*_*t*_ is the number of new infections at time *t*, and $\overset{t}{\underset{s=1}{\sum }}I_{t-s}\;w_{s}$ is the sum of number of infections up to time *t – s*, weighted by the infectivity function *w*_*s*_. The latter is approximated by the probability distribution of the serial interval (namely the time between successive cases in a chain of transmission). We sampled the serial interval from a family of Gamma distributions with mean 4.6 days (95% Credible Intervals (CrI): 3.7, 6.0) and standard deviation 2.9 days (95% CrI: 1.9, 4.9), as recently observed in China (11). *R*_*t*_ estimates were then smoothed using a 7-day time window.

### Modeling of the Epidemic Behavior and Short-Term Forecast

We analyzed the daily count of new infections using two phenomenological models:

(i) the generalized logistic growth model (GLM), which extends the simple logistic growth model to accommodate sub-exponential growth dynamics with a scaling of the growth parameter, *p* (6):
  $$\begin{array}{l} C\prime (t)=rC{\left(t\right)}^{p}\left[1-\frac{C(t)}{K}\right] \end{array}$$
  where *C*′(*t*) is incidence growth phase over time *t*, *C*(*t*) is the cumulative number of cases at time *t, r* is the intrinsic growth rate in the absence of any control, *p* is a scaling of growth parameter, ranging from 0 (constant incidence) to 1 (exponential growth), and *K* is the final size of the epidemic;
(ii) the generalized modified Richards model (GRM), which allows departures from the S-shaped dynamics of the classical logistic growth model, and incorporates the possibility of growth deceleration (12, 13):
  $$\begin{array}{l} C\prime (t)=rC{\left(t\right)}^{p}\left[1-{\left(\frac{C(t)}{K}\right)}^{a}\right] \end{array}$$
  where *a* is the deviation from the S-shaped dynamics of the logistic growth model.

Both models were fitted to data in order to characterize the pattern of the epidemic in its early phases, produce 5 days forecast of the number of new infections, and estimate the peak time and the final size of the epidemic curve. Both models allow for estimation of uncertainly, based on bootstrap resampling.

## Results

**Figure 1:**
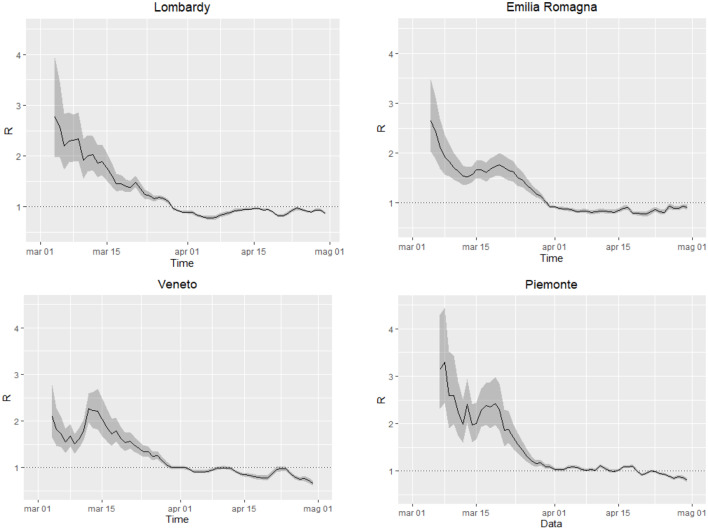
Time-dependent reproduction number *R*_*t*_ in the regions Lombardy, Veneto, Emilia Romagna and Piedmont, from March 3rd to April 30th. Black solid line: estimate of *R*_*t*_, gray areas: 95% confidence intervals, dotted line: threshold for outbreak extinction.

*R*_*t*_ has decreased over time in all regions, reaching estimates below 1.0 (Figure 1), the threshold under which the epidemic dies out, at the beginning April in Lombardy, Emilia-Romagna, and Veneto and at the end of April in Piedmont. In all regions, *R*_*t*_ started from values ranging between 2.0 and 3.0, consistent with estimates obtained in other contexts (14). In Veneto, the steep increase on March 12th likely reflects changes (increases) in the testing practices (between March 10th and March 11th the daily number of tests increased by 28%; previously, the daily average increase was 7%). The level of uncertainty decreases over time, with the increasing number of events.

**Figure 2:**
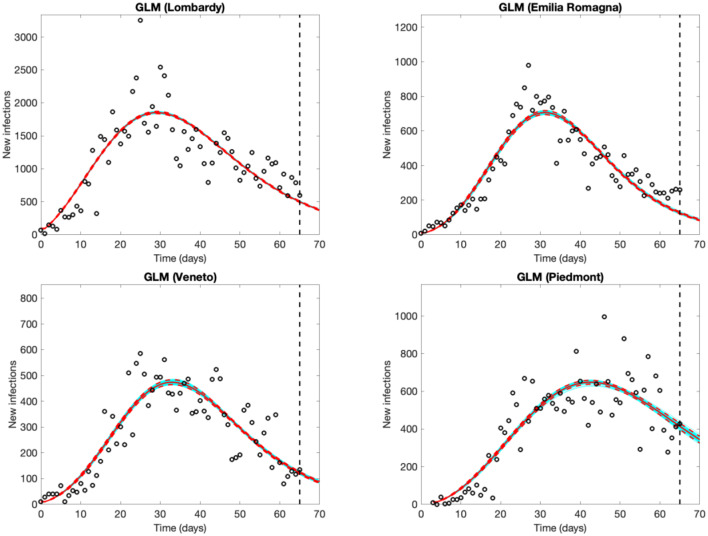
Five-day Generalized Logistic Model (GLM) forecasts of SARS-CoV-2 new infections in Lombardy, Emilia Romagna and Veneto (observed data: Feb. 25th to April 30th), and Piedmont (observed data: Feb. 28th to April 30th). Empty circles represent new observed cases, the vertical dashed line indicates where the real observations stop, the red continuous line the best prediction of the epidemic in the following 5 days, the red dashed lines the 95% confidence bands, and the blue lines the bundle of models estimated by the prediction algorithm. Bootstrap size was set to 100.

The four regions experienced an increasing number of observed new cases until March 25–26 in Lombardy, until a couple of days later in Emilia Romagna and Veneto, and until 12–14 days later in Piedmont, well-captured by the models. Forecasts from the GLM (Figure 2) and GRM models (Figure S1 in Supplementary Material) are very similar, supporting their reliability. Results are also consistent with the decrease of *R*_*t*_. The estimates of the final epidemic size predicted on April 30th range from 84,000 (GRM) to 85,000 cases (GLM) in Lombardy, 35,000 (GLM) to 37,000 (GRM) in Piedmont, 27,000 (both GLM and GRM) in Emilia Romagna, 20,000 (both GLM and GRM) in Veneto. All parameter estimates with their 95% confidence intervals are shown in Table S1.

The daily variation may be large, especially in the earlier phases of the epidemic, and strongly affected by variations over time in testing practices and, possibly, reporting. The uncertainty is larger, as expected, when using the more flexible GRM model. Large daily variations in forecasts are observable in Figure S2, showing consecutive 5-days forecasts of new cases in Lombardy, from March 22nd to March 29th, in the week when the epidemic curves reached the peak.

**Figure 3:**
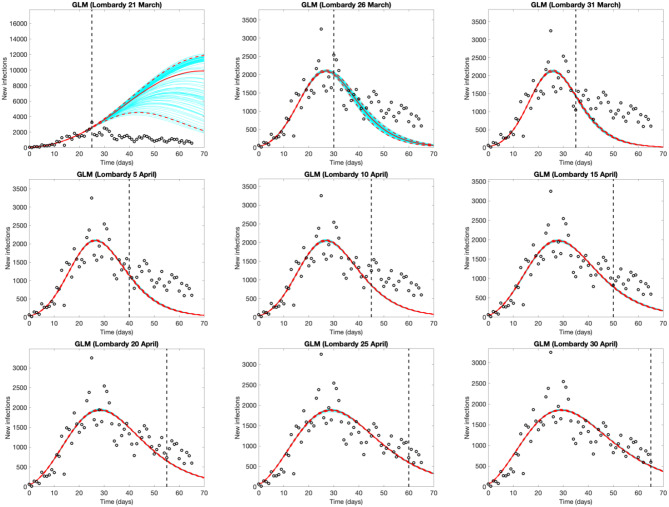
Evolution of the epidemic predictions in Lombardy based on the Generalized Logistic Model (GLM). An increasing amount of epidemic data (black circles) are used, starting from Feb. 25th until March 21st (day of the total lockdown) and then extending the data by 5 days until April 30th. Empty circles represent observed cases, the vertical dashed line indicates where the real observations stop, the red continuous line the best prediction of the epidemic up to May 5th (day 70 of the epidemic), the red dashed lines the 95% confidence bands, and the blue lines the bundle of models estimated by the prediction algorithm. Bootstrap size was set to 100.

Figure 3 shows the evolution of the epidemic forecasts in Lombardy with an increasing number of observed data, starting from the day of the lockdown (March 21st, day 25 of the epidemic). The first graph shows that on March 21st, the GLM predicts a sub-exponential growth but 5 days later it identifies the peak and predicts an over-optimistic decline. GLM predictions start appearing reasonable after mid-April, when the model captures a decline that appears much slower than the initial rise. Epidemic evolution in Emilia Romagna, Veneto and Piedmont are shown in Figures S3–S5, respectively.

**Figure 4:**
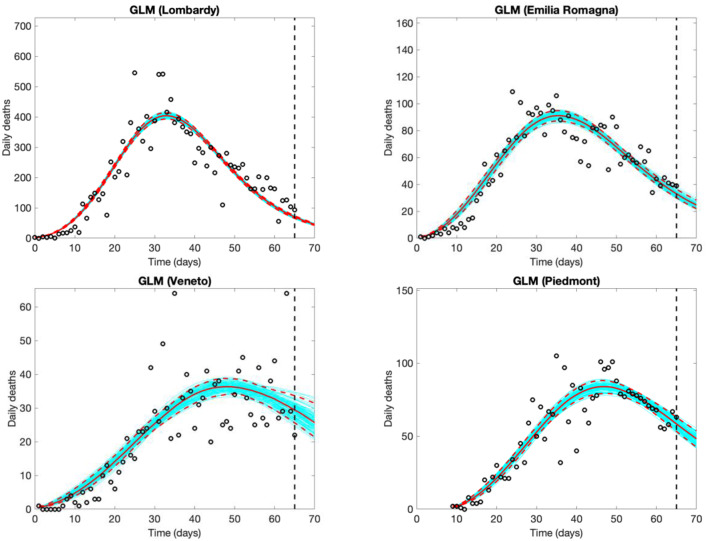
Five-day Generalized Logistic Model (GLM) forecasts of SARS-CoV-2 deaths in Lombardy (observed data: Feb. 25th to April 30th), Veneto and Emilia Romagna (observed data: Feb. 26th to April 30th), and Piedmont (observed data: March 5th to April 30th). Empty circles represent deaths, the vertical dashed line indicates where the real observations stop, the red continuous line the best prediction of the epidemic in the following 5 days, the red dashed lines the 95% confidence bands, and the blue lines the bundle of models estimated by the prediction algorithm. Bootstrap size was set to 100.

Estimated time trends and 5-day forecasts for daily COVID-19 deaths should theoretically follow, by ~1–15 days, the trends of new cases, and are thus less informative for decision making, but are possibly less affected by testing and reporting variations (Figure 4, results from the GLM model only). Due to the smaller numbers, the uncertainty in the models for both the observed shape of the epidemic and the 5-day forecast is larger for the number of deaths than for the number of new cases.

## Discussion

In this study, we applied empirical models to daily COVID-19 incident cases, in the four Italian regions most affected by the outbreak, as April 30th.

We observed an almost monotonic decrease of the estimates of *R*_*t*_ in all four regions and a decrease of incident cases starting approximately from March 25th in Lombardy, a few days later in Emilia Romagna and Veneto, and a dozen of days later in Piedmont. These findings may reflect the effects of the lockdown, that start being appreciable after ~2 weeks. These results are consistent with what observed in Wuhan Province, China (WHO, 2020^1^). The monitoring of *R*_*t*_ provides a useful tool to describe the real-time epidemic strength and to capture potential impact of the implemented control measures. Our results suggest that costly and disruptive public health controls have been effective in limiting the Sars-Cov-2 spread in Northern Italy, as suggested by other studies (15, 16, 19) and may support to the implementation of similar policies in other countries.

We suggest that reporting of daily updated *R*_*t*_ estimates and applying GLM and/or GRM to observed data may complement more common approaches used to monitor SARS-CoV-2 epidemics in its early phases. The same approach may be used also in areas less affected by the epidemic but potentially at risk, such as several regions in the Centre and South of Italy (17). These phenomenological models are relatively easy to implement and offer opportunities to monitor the positive impact of measures introduced to control the epidemic, characterize the pattern of the epidemic both in its early and late phases, produce short-term forecasts and estimate the peak time and the final size of the epidemic curve. Whereas, short-term (e.g., 5 days) predictions can be interpreted and used to make timely decisions as the outbreak proceeds, long-term predictions of the epidemic are interpretable only after the peak of the epidemic has been reached, as observed when phenomenological models were fitted at different time-steps (Figure 4).

Being empirical, these approaches are affected by testing and reporting changes over time. However, this limitation is potentially common to the majority of models, both mechanistic and empirical, given that they rely on reported data for the estimation or calibration phase. This limitation should be considered when interpreting the results and forecasts. For instance, *R*_*t*_ estimates are influenced by the variation over time of testing policies and thus the probability of identifying new cases. This, for example, can be appreciated in the temporary overestimation of *R*_*t*_ observed in Veneto around the 12th of March (Figure 1), when the number of tests abruptly increased Short-term forecasts provided by GLM and/or GRM may change every day, as the number of reported cases fluctuate, influencing prediction, especially in the early phases of an outbreak. The more flexible (and quick to capture variations) the model is, the stronger the variation. It is therefore essential to consider the full range of uncertainty, as well as to revise the predictions on a daily basis. Taking this into account, forecast models yield a good visual fit to the epidemic curves, and the estimated parameters (Supplementary Material) can be interpreted in terms of describing the epidemic dynamics. Like *R*_*t*_, also GLM and GRM forecasts rely on reported data and are affected by under-reporting. However, taking this limitation into account, their application can help describing and interpreting the epidemic evolution. For instance, Lombardy experienced a slower decrease of daily infection than those predicted by GLM (Figure 3). This could be explained as an intrinsic pattern of the epidemic curve or as results of a higher testing capacity in the late phase of the epidemic.

In conclusion, our study suggests that timely indications for public health authorities and governments are essential to slow down the epidemic and release the pressure on overburdened health systems. Models applied in this study may help in underlining early signs of the success of costly and disruptive public health controls and reinforce the idea that collective efforts are working, are vital to “hold the line” and should not be abandoned prematurely.

## Data Availability Statement

Publicly available datasets were analyzed in this study. This data can be found here: https://github.com/pcm-dpc/COVID-19/tree/master/dati-regioni.

## Ethics Statement

The study was based on publicly available aggregate data. No Ethics committee approval was necessary.

## Author Contributions

All authors conceived the study, carried out the statistical analysis and drafted the final version of the manuscript.

## Conflict of Interest

The authors declare that the research was conducted in the absence of any commercial or financial relationships that could be construed as a potential conflict of interest.

## Footnotes

^1^Available online at: https://www.who.int/docs/default-source/coronaviruse/who-china-joint-mission-on-COVID-19-final-report.pdf (accessed March 29, 2020).
