# Supplementary material for: Approaches to Daily Monitoring of the SARS-CoV-2 Outbreak in Northern Italy
Source: Front Public Health. 2020 May 22;8:222. doi: 10.3389/fpubh.2020.00222 (PMC7256452; doi:10.3389/fpubh.2020.00222)
Supplement: Supplementary file 1 [file Data_Sheet_1.docx]

**Table S1**. Five-days GLM and GRM forecasts of SARS-CoV-2 new infections in Lombardy, Emilia Romagna and Veneto (observed data: Feb 25^th^ to April 30^th^), and Piedmont (observed data: Feb 28^th^ to April 30^th^).

| **Region** | **GLM** | | **GRM** | | |
| --- | --- | --- | --- | --- | --- |
| **Region** | **Parameters** | **Estimates (95% CIs)** | **Parameters** | **Estimates (95% CIs)** |  |
| **Lombardy** | *r*  *p*  *K (10^-5^)* | 5.30 (95% CI: 5.10, 5.60)  0.61 (95% CI: 0.61, 0.62)  0.84 (95% CI: 0.83, 0.85) | *r*  *p*  *K (10^-5^)*  *a* | 2.60 (95% CI: 2.40, 3.10)  0.72 (95% CI: 0.69, 0.73)  0.85 (95% CI: 0.84, 0.86)  0.53 (95% CI: 0.50, 0.65) |  |
| **Emilia Romagna** | *r*  *p*  *K (10^-5^)* | 1.80 (95% CI: 1.70, 1.90)  0.70 (95% CI: 0.69, 0.70)  0.27 (95% CI: 0.26, 0.27) | *r*  *p*  *K (10^-5^)*  *a* | 1.10 (95% CI: 0.99, 1.20)  0.81 (95% CI: 0.77, 0.82)  0.27 (95% CI: 0.26, 0.27)  0.53 (95% CI: 0.50, 0.67) |  |
| **Veneto** | *r*  *p*  *K (10^-5^)* | 1.60 (95% CI: 1.50, 1.70)  0.69 (95% CI: 0.68, 0.70)  0.20 (95% CI: 0.19, 0.20) | *r*  *p*  *K (10^-5^)*  *a* | 1.00 (95% CI: 0.90, 1.30)  0.79 (95% CI: 0.74, 0.81)  0.20 (95% CI: 0.20, 0.20)  0.56 (95% CI: 0.50, 0.77) |  |
| **Piedmont** | *r*  *p*  *K (10^-5^)* | 2.20 (95% CI: 2.10, 2.40)  0.65 (95% CI: 0.63, 0.66)  0.36 (95% CI: 0.35, 0.37) | *r*  *p*  *K (10^-5^)*  *a* | 1.50 (95% CI: 1.40, 1.90)  0.72 (95% CI: 0.68, 0.74)  0.37 (95% CI: 0.36, 0.39)  0.56 (95% CI: 0.50, 0.79) |  |

**Fig. S1.** Five-day Generalized Richard’s Model forecasts of SARS-CoV-2 new infections in Lombardy, Emilia Romagna and Veneto (observed data: Feb 25^th^ to April 30^th^), and Piedmont (observed data: Feb 28^th^ to April 30^th^).

| 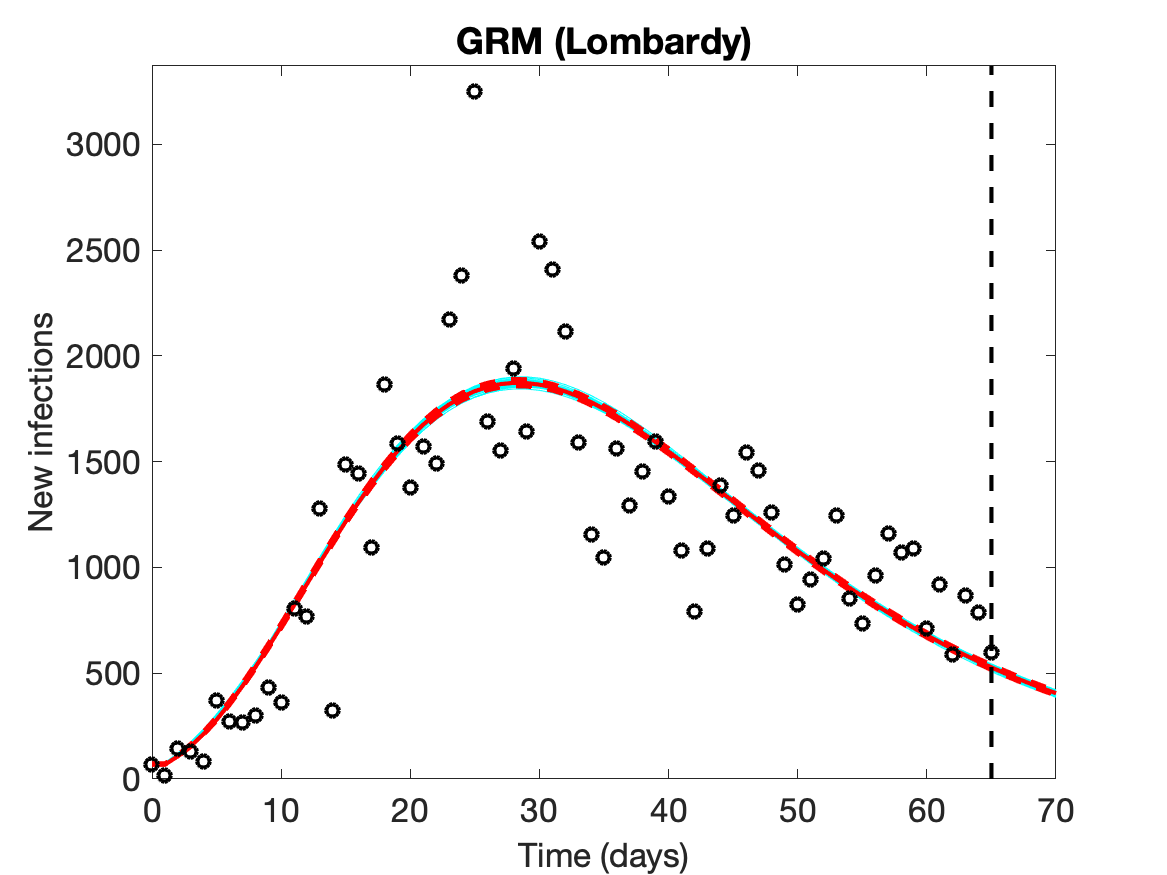 | 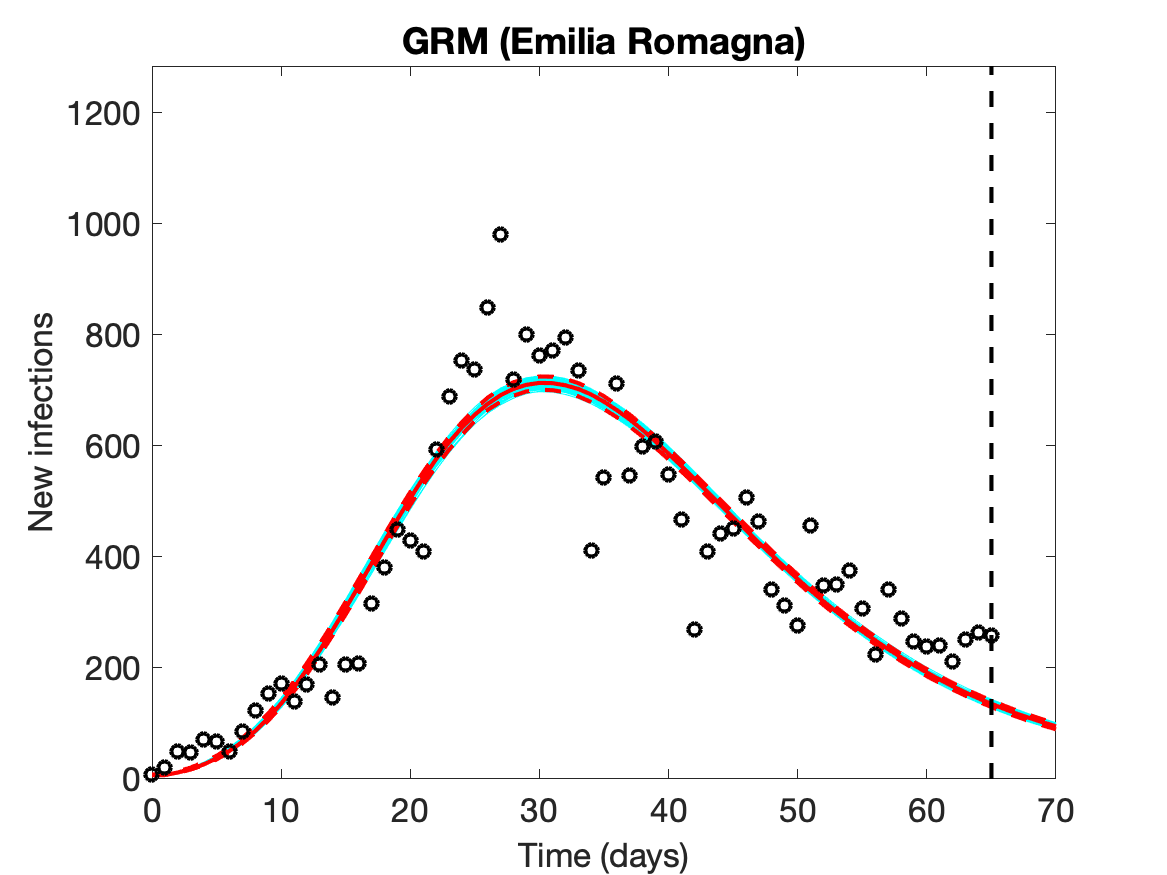 |
| --- | --- |
| 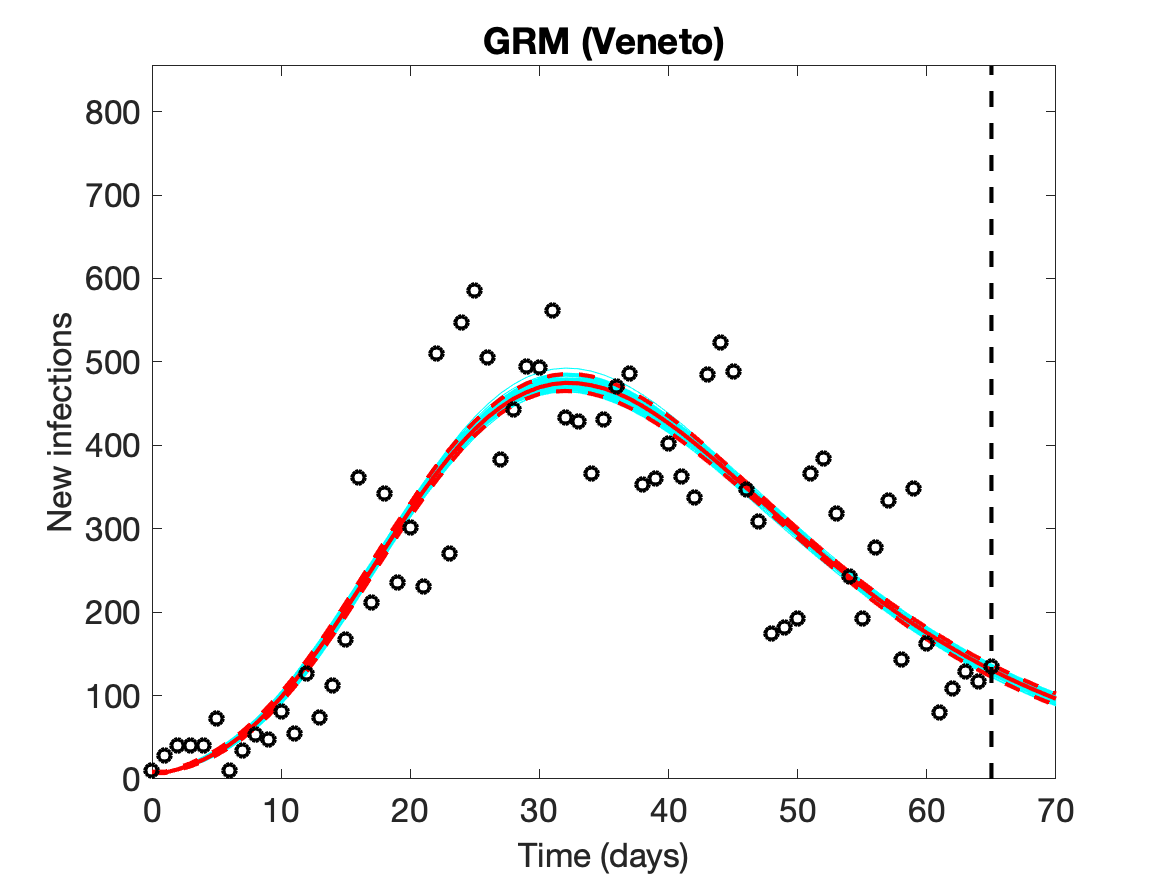 | 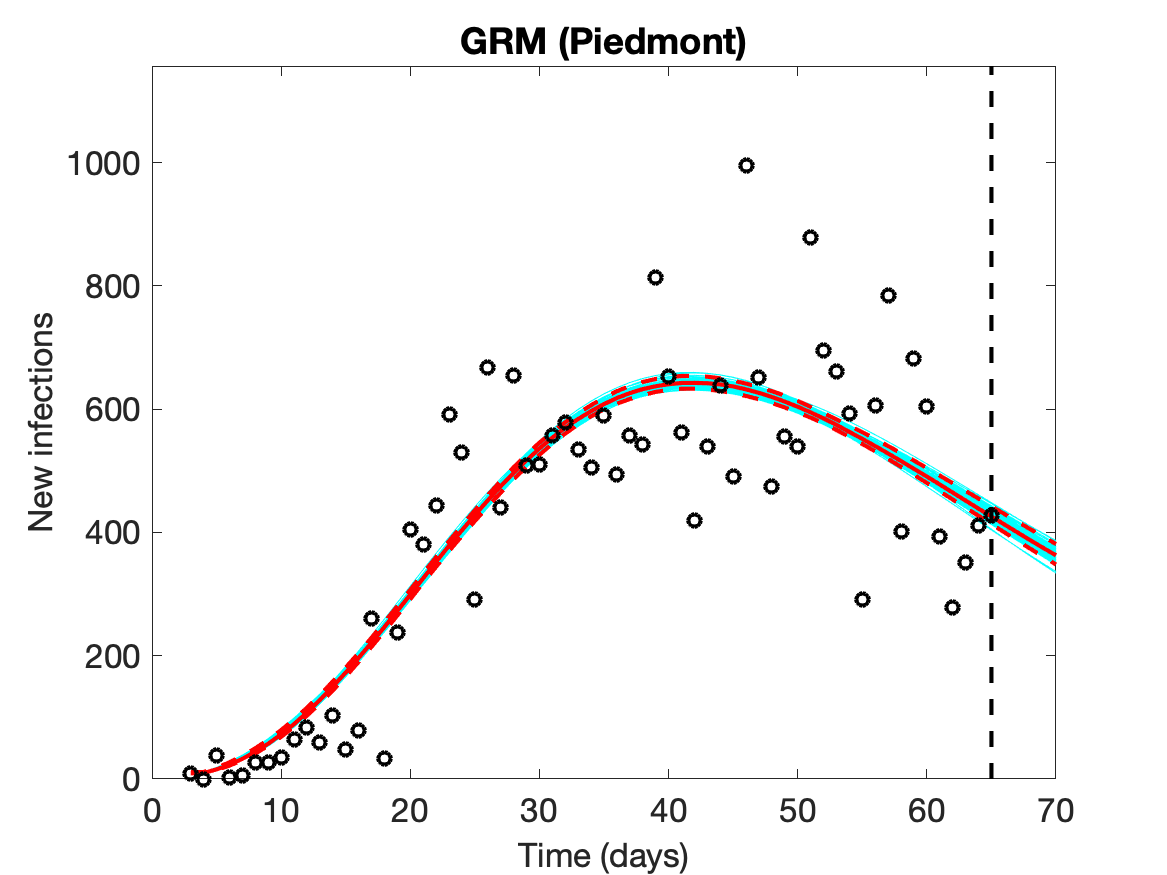 |

Empty circles represent new observed cases, the vertical dashed line indicates where the real observations stop, the red continuous line the best prediction of the epidemic in the following 5 days, the red dashed lines the 95% confidence bands, and the blue lines the bundle of models estimated by the prediction algorithm. Bootstrap size was set to 100.

**Fig. S2.** Consecutive five-days GLM forecasts of SARS-CoV-2 new infections in Lombardy (observed data: Feb 28^th^ - March 20^th^ up to Feb 28^th^ – March 29th).

| **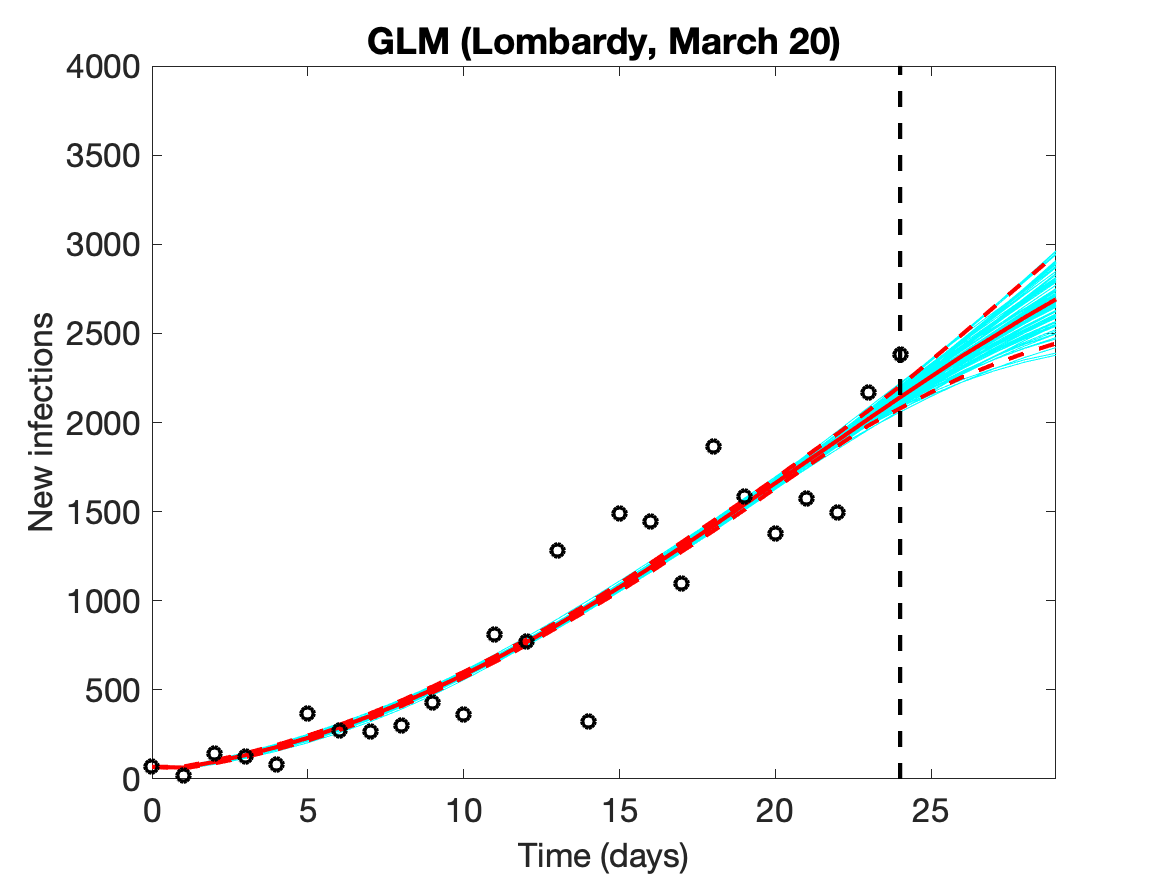** | **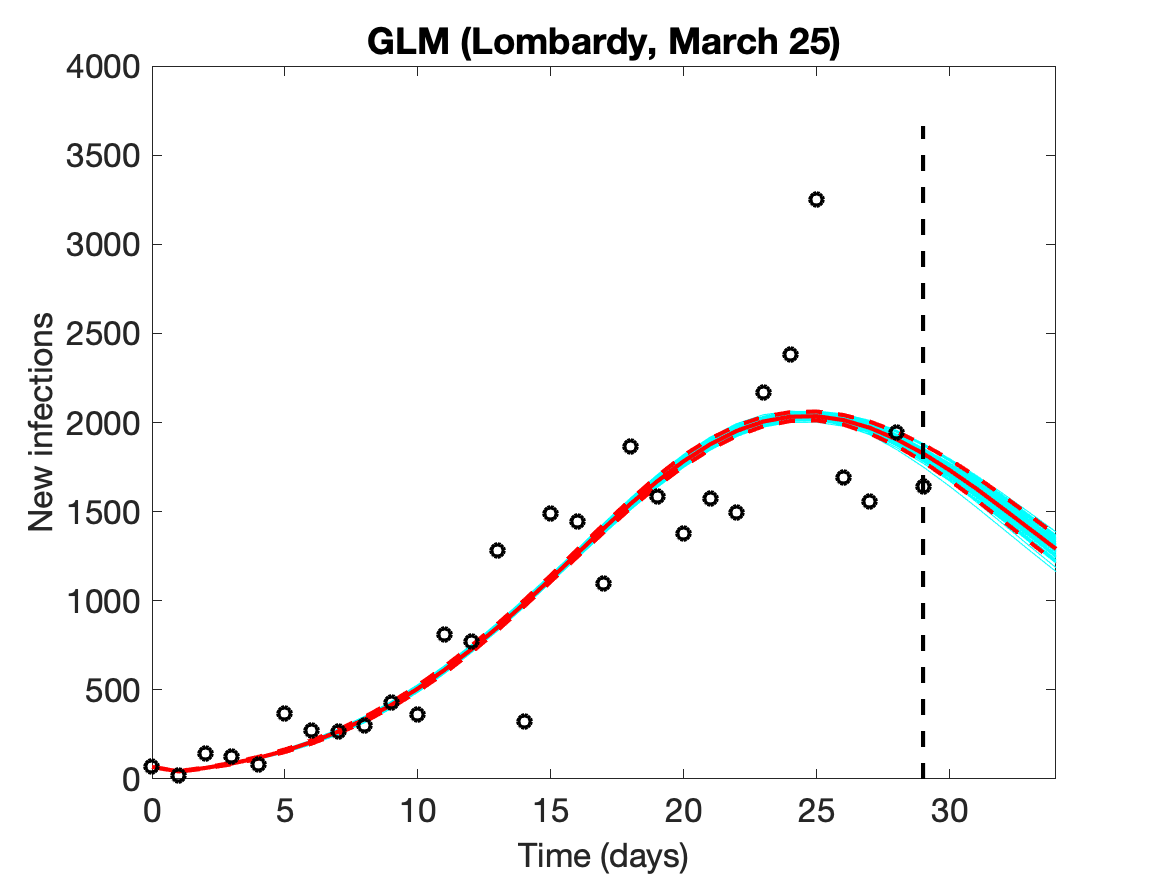** |
| --- | --- |
| **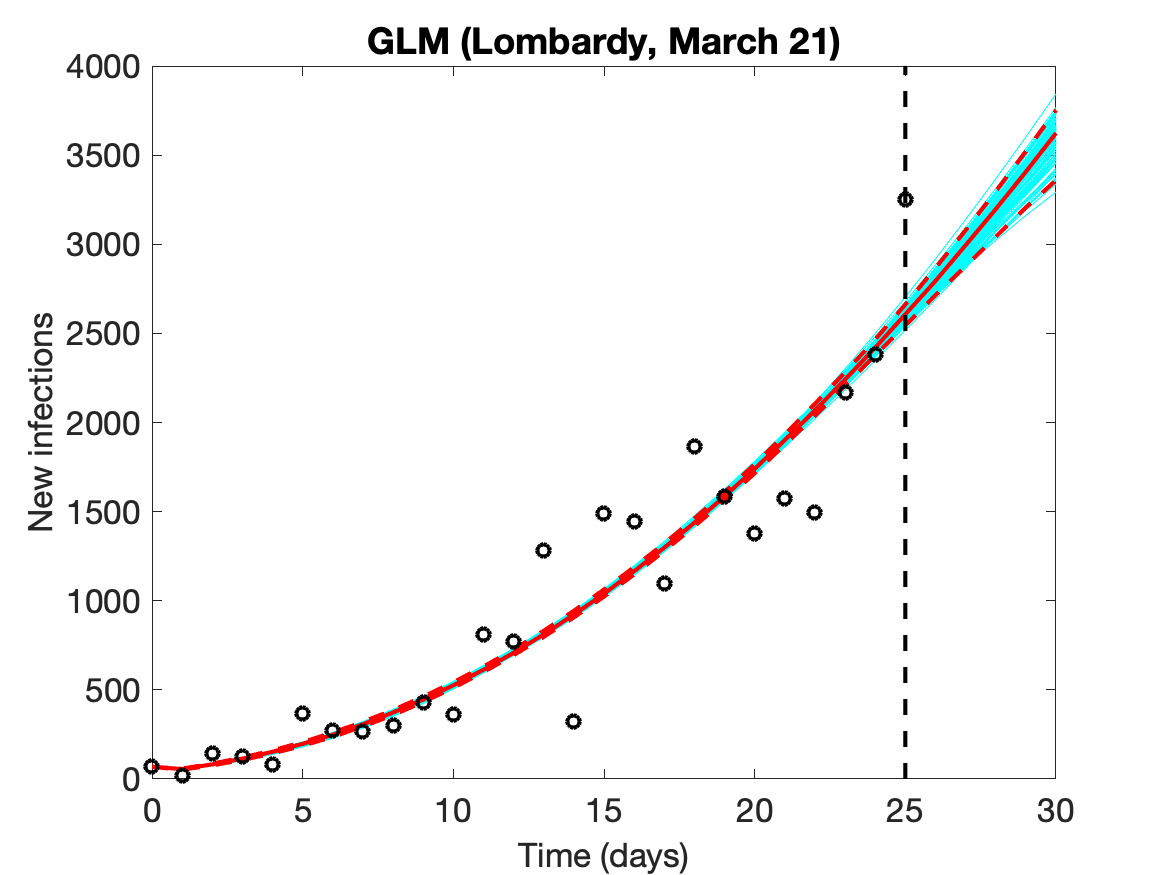** | **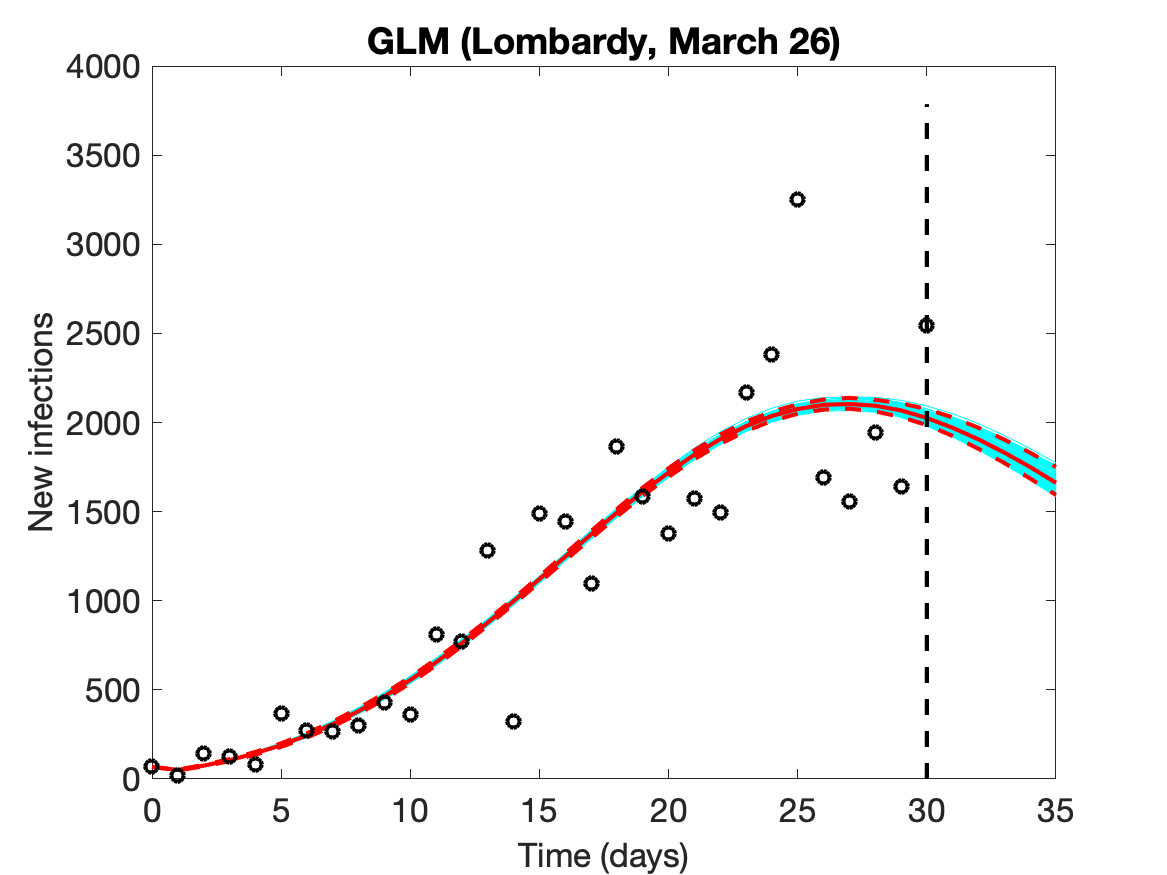** |
| **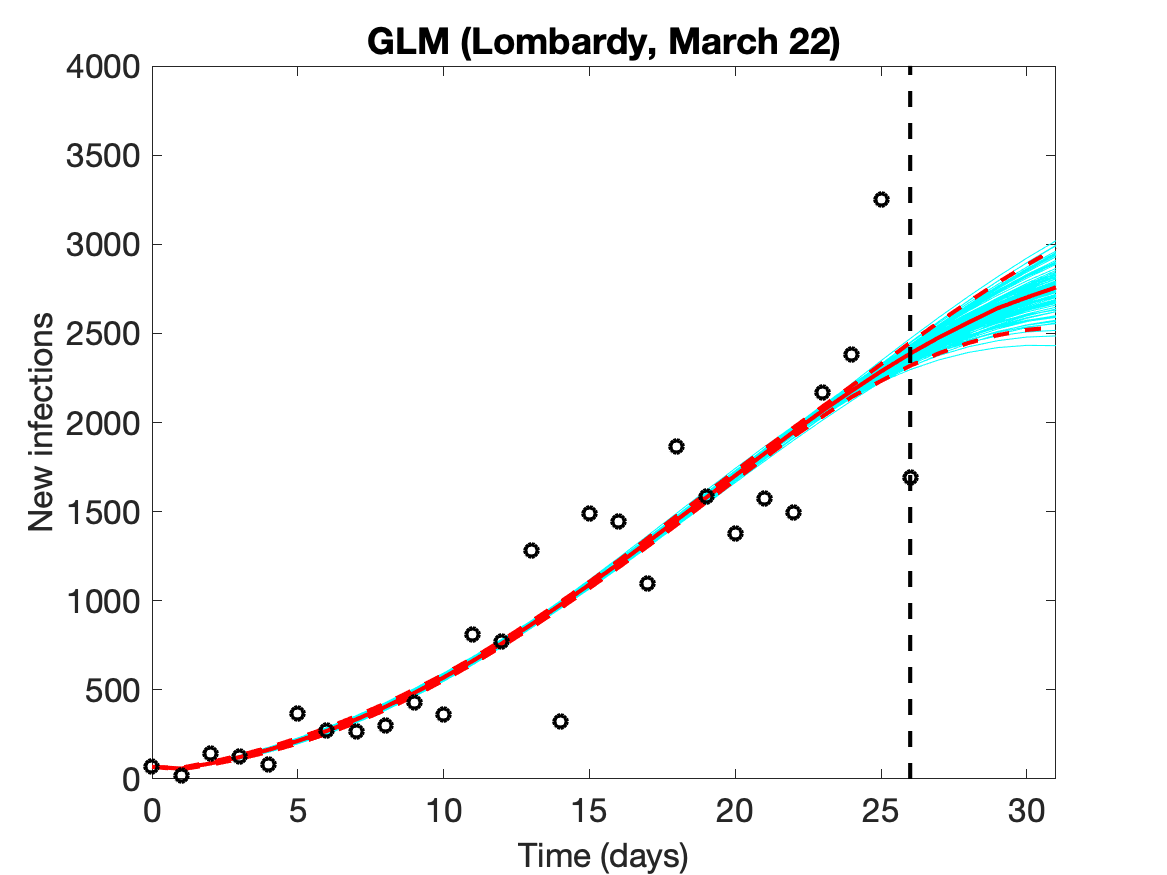** | **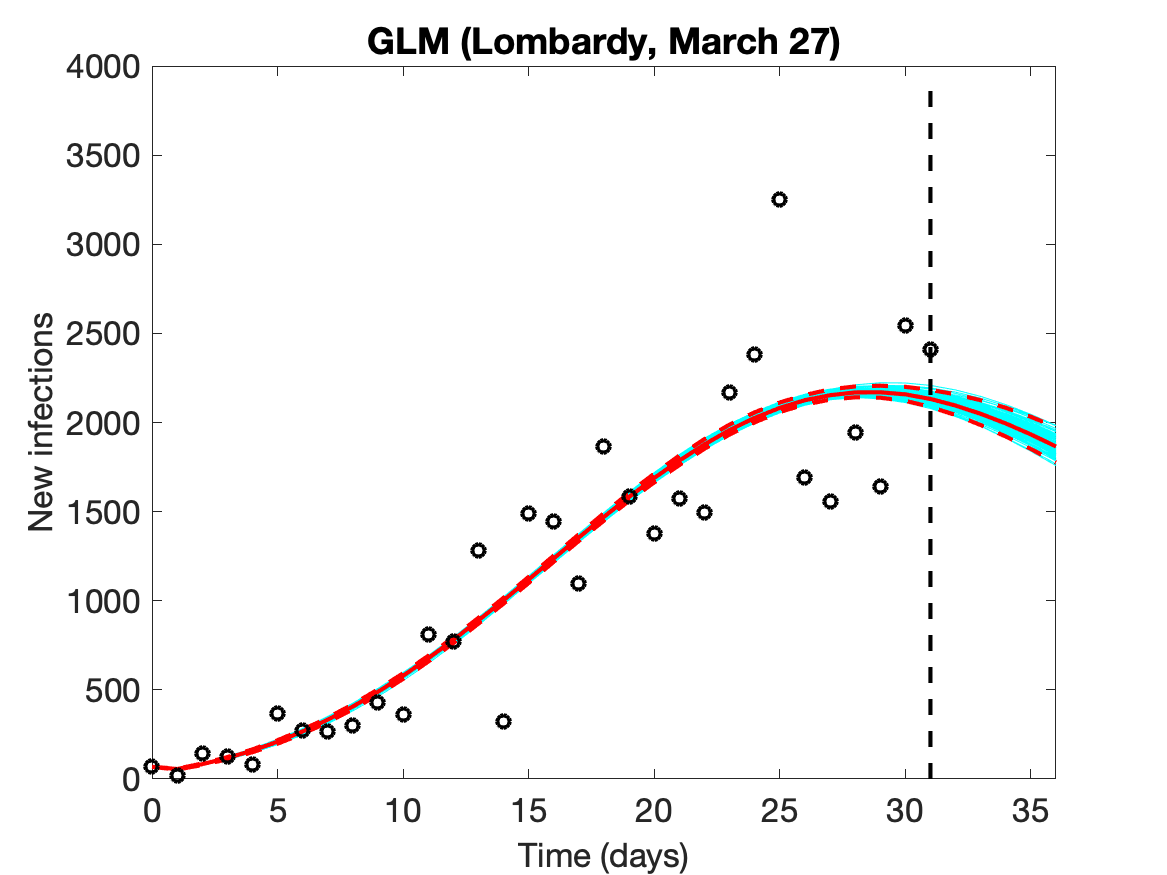** |
| **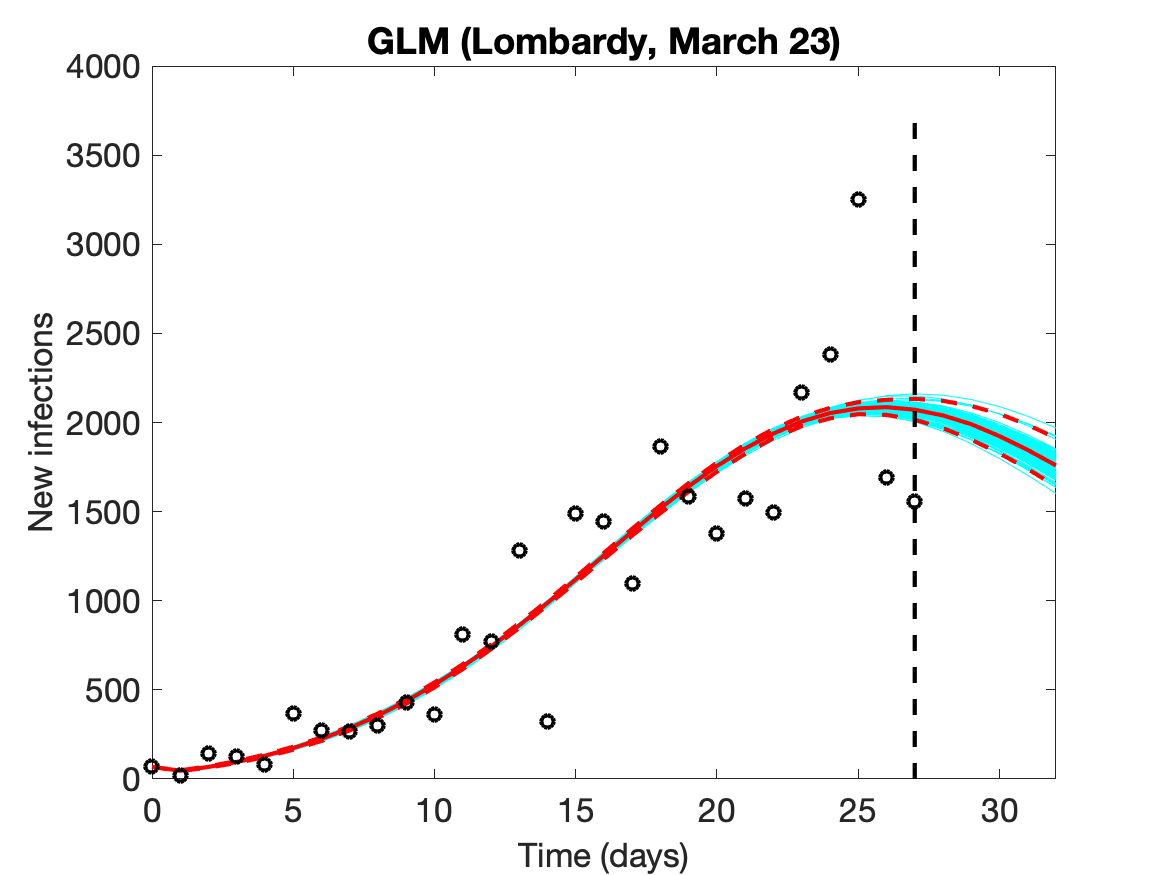** | **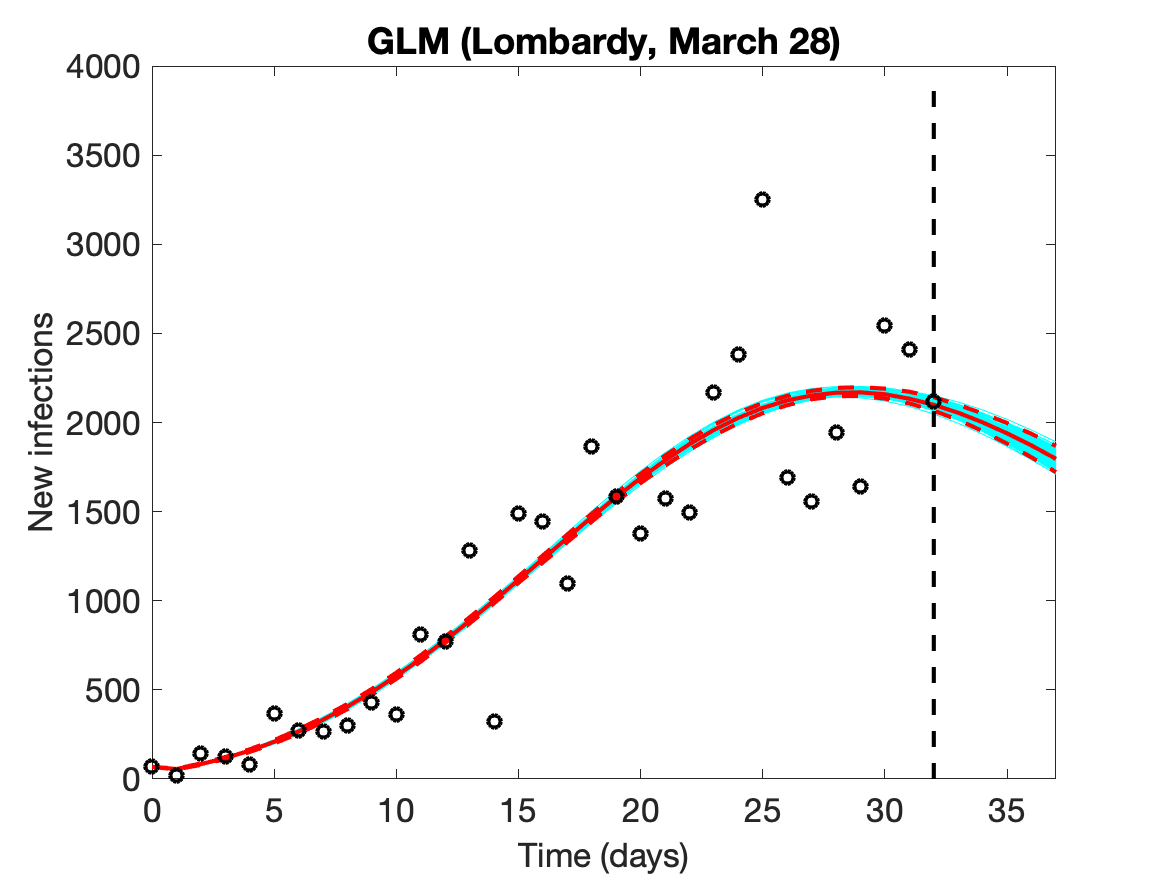** |
| **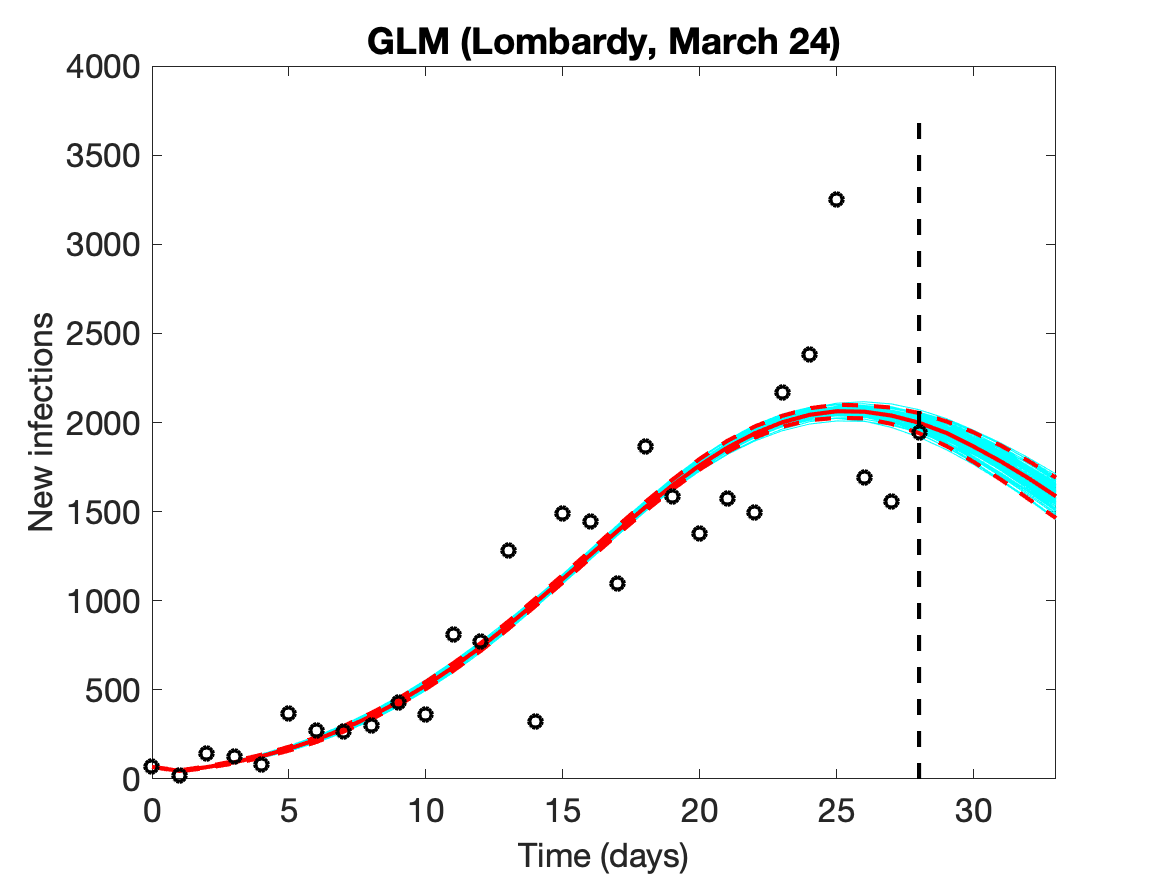** | **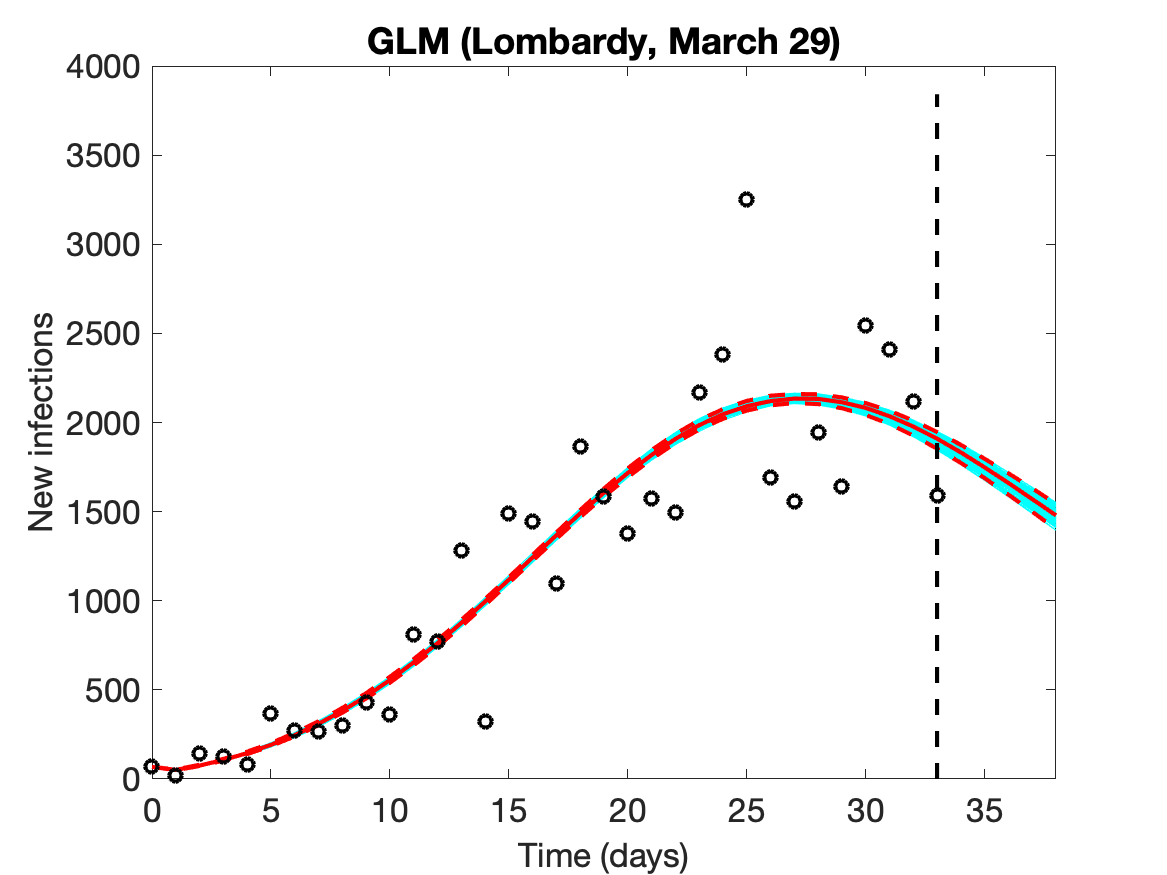** |

**Fig. S3.** Evolution of the epidemic predictions in Emilia Romagna based on the Generalized Logistic Model (GLM). An increasing amount of epidemic data (black circles) are used, starting from Feb 25^th^ until March 21^st^ (day of the total lockdown) and then extending the data by 5 days until April 30^th^.

| **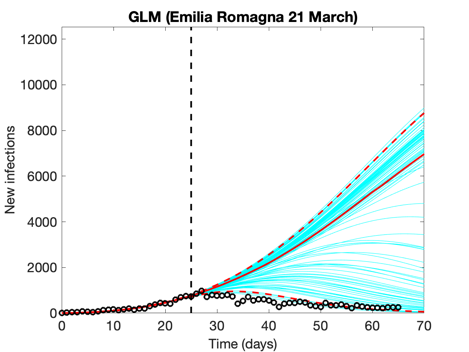** | **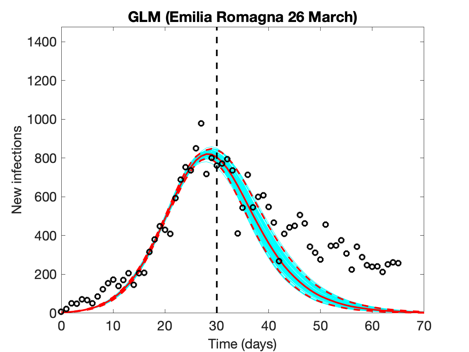** | **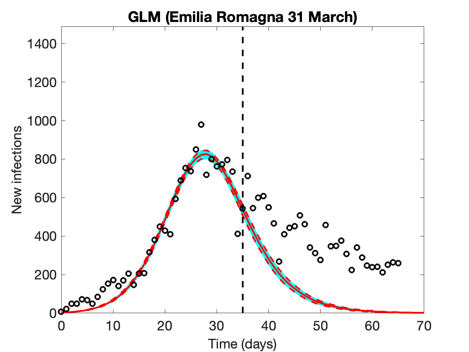** |
| --- | --- | --- |
| **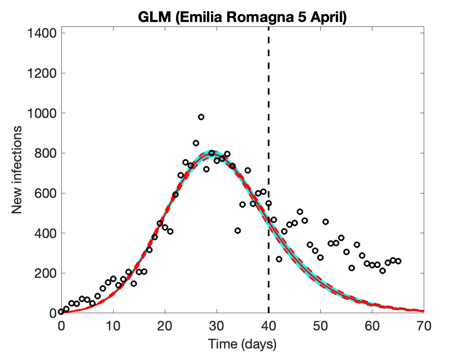** | **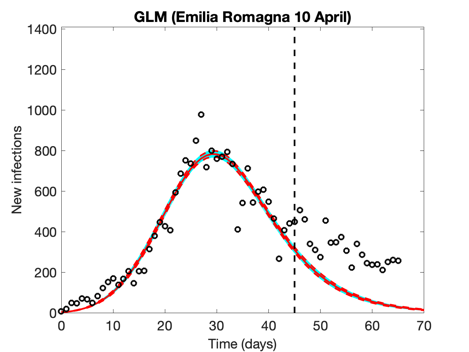** | **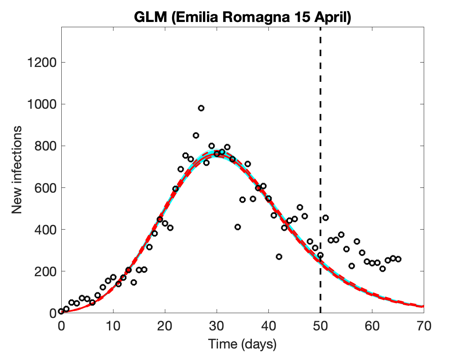** |
| **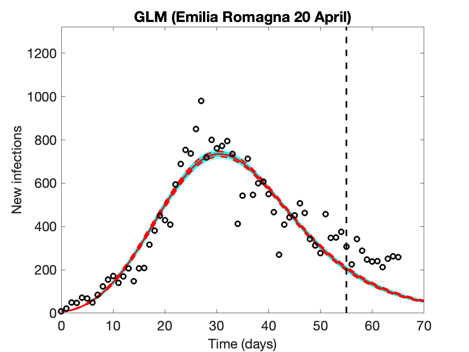** | **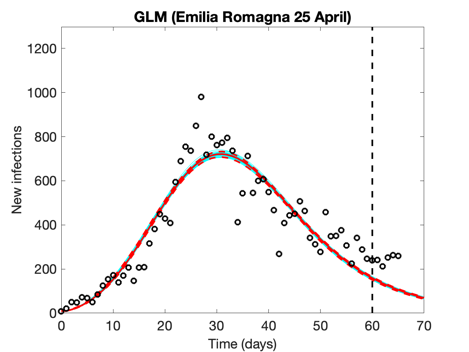** | **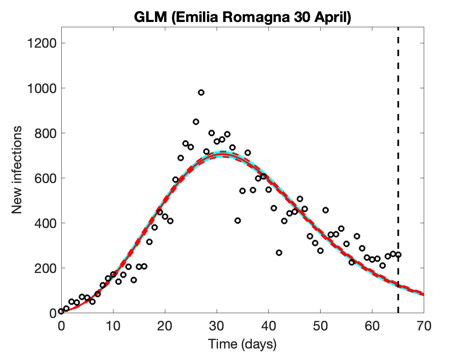** |

Empty circles represent observed cases, the vertical dashed line indicates where the real observations stop, the red continuous line the best prediction of the epidemic up to May 5^th^ (day 70 of the epidemic), the red dashed lines the 95% confidence bands, and the blue lines the bundle of models estimated by the prediction algorithm. Bootstrap size was set to 100.

**Fig. S4.** Evolution of the epidemic predictions in Veneto based on the Generalized Logistic Model (GLM). An increasing amount of epidemic data (black circles) are used, starting from Feb 25^th^ until March 21^st^ (day of the total lockdown) and then extending the data by 5 days until April 30^th^.

.

| **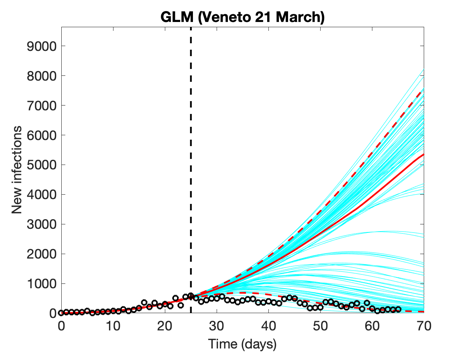** | **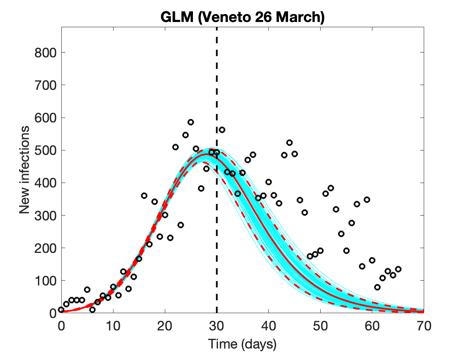** | **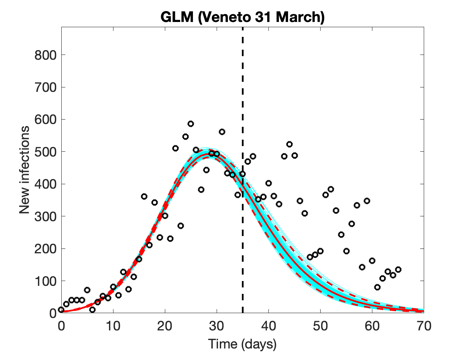** |
| --- | --- | --- |
| **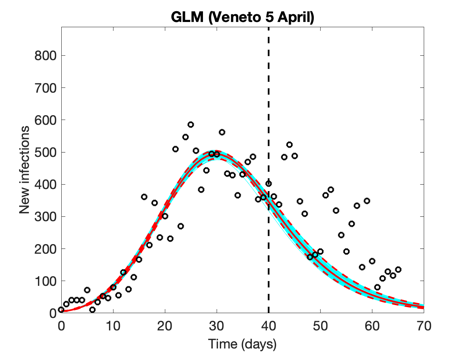** | **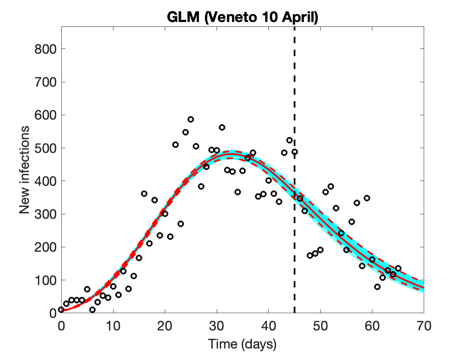** | **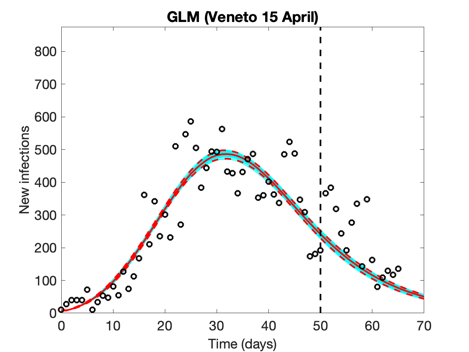** |
| **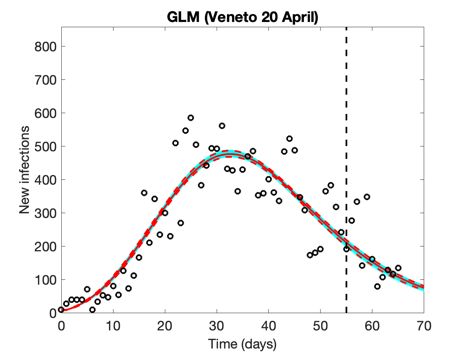** | **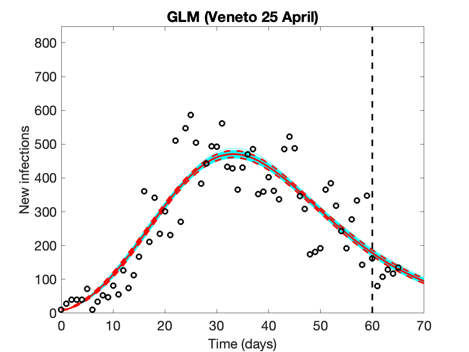** | **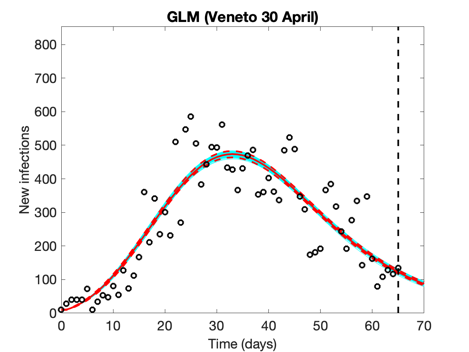** |

Empty circles represent observed cases, the vertical dashed line indicates where the real observations stop, the red continuous line the best prediction of the epidemic up to May 5^th^ (day 70 of the epidemic), the red dashed lines the 95% confidence bands, and the blue lines the bundle of models estimated by the prediction algorithm. Bootstrap size was set to 100.

**Fig. S5.** Evolution of the epidemic predictions in Piedmont based on the Generalized Logistic Model (GLM). An increasing amount of epidemic data (black circles) are used, starting from Feb 28^th^ until March 21^st^ (day of the total lockdown) and then extending the data by 5 days until April 30^th^.

| **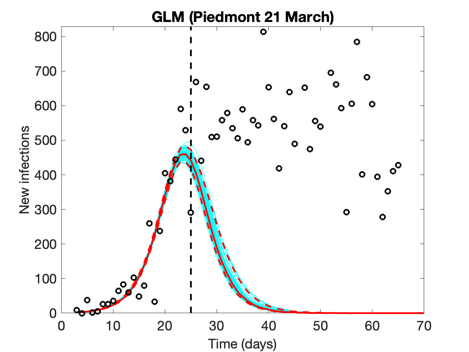** | **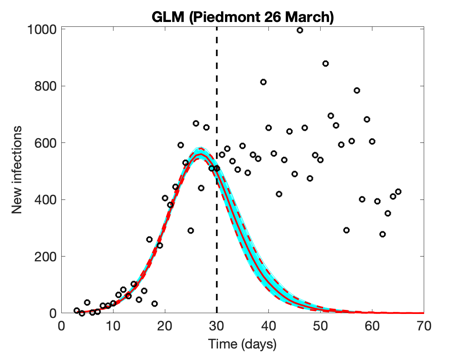** | **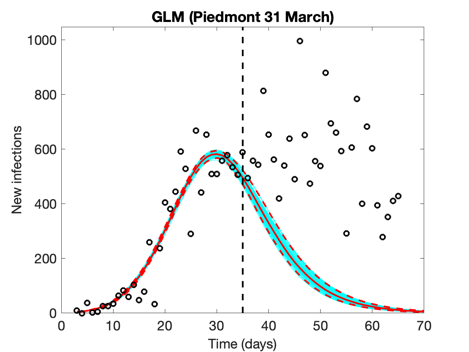** |
| --- | --- | --- |
| **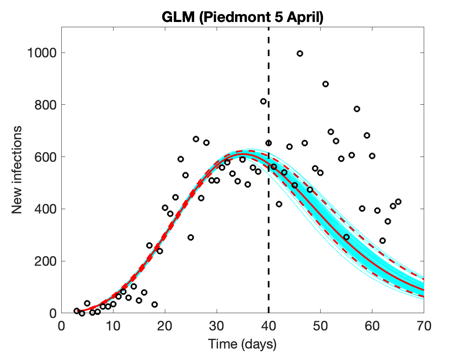** | **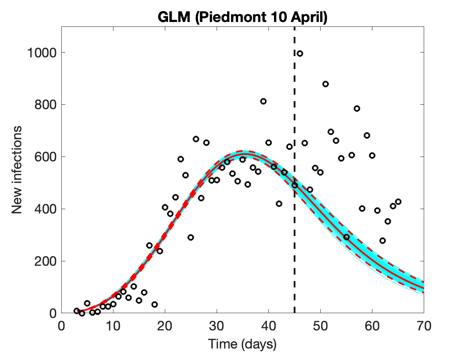** | **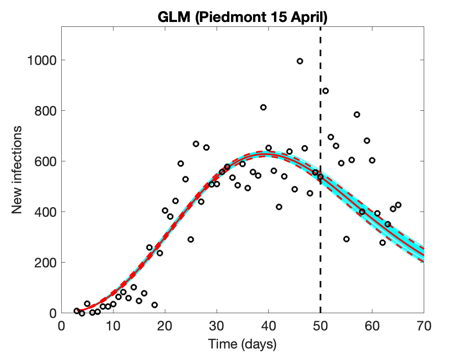** |
| **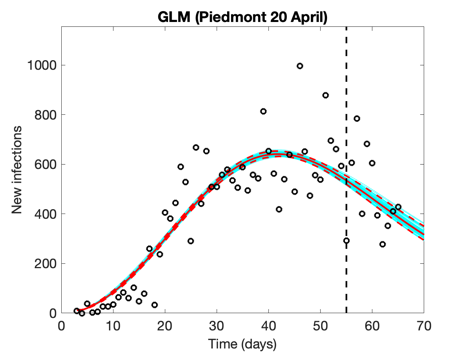** | **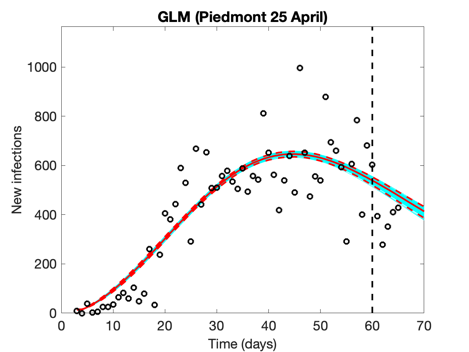** | **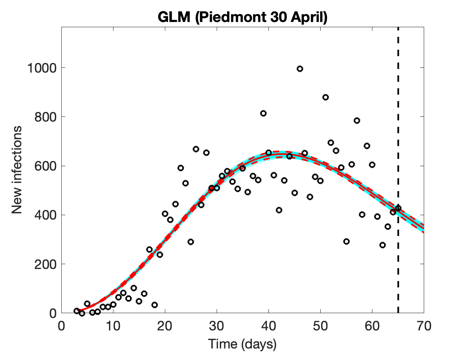** |

Empty circles represent observed cases, the vertical dashed line indicates where the real observations stop, the red continuous line the best prediction of the epidemic up to May 5^th^ (day 70 of the epidemic), the red dashed lines the 95% confidence bands, and the blue lines the bundle of models estimated by the prediction algorithm. Bootstrap size was set to 100.
